# The impact of a brief online education intervention on the knowledge, attitudes and beliefs of qualified physiotherapists about chronic low back pain: Pre- and post-intervention survey

**DOI:** 10.4102/sajp.v82i1.2264

**Published:** 2026-05-29

**Authors:** Lara Davis, Benita Olivier, Lorraine Jacobs, Sandy Lord

**Affiliations:** 1 Department of Physiotherapy, Faculty of Health Sciences, University of the Witwatersrand, Johannesburg, South Africa; 2 Centre for Healthy Living Research, Oxford Institute of Applied Health Research, Faculty of Health, Science and Technology, Oxford-Brookes University, Oxford, United Kingdom; 3 Physios in Touch, Private Practice, Johannesburg, South Africa

**Keywords:** attitudes, beliefs, pain knowledge, low back pain, chronic, physiotherapist, education, risk stratification

## Abstract

**Background:**

A shift from biomedical towards biopsychosocial approaches is increasingly recommended in literature for the management of non-specific low back pain (LBP). The knowledge, attitudes and beliefs of clinicians may, however, serve as a barrier to effective management.

**Objectives:**

Our study investigated the impact of an online education programme on the knowledge, attitudes and beliefs of physiotherapists about chronic LBP.

**Method:**

Qualified physiotherapists (*n* = 536) attended a webinar series based on risk stratification and the biopsychosocial model of care for LBP. Participants completed the Pain Attitudes and Beliefs Scale for Physiotherapists and Neurophysiology of Pain Questionnaire before and after. Data were compared using descriptive analysis, dependent *t*-tests and Pearson correlation analyses.

**Results:**

Post-intervention, biomedical orientation decreased significantly (53.74 ± 12.80 to 43.04 ± 11.63; *p* < 0.001), while biopsychosocial orientation increased (35.52 ± 4.65 to 39.62 ± 4.70; *p* < 0.001). Pain neurophysiology knowledge improved significantly (NPQ: 10.19 ± 1.78 to 11.04 ± 1.64; *p* < 0.001). Improvements in the biopsychosocial orientation of participants were found to correlate with a notable reduction in biomedical orientation post-intervention (*r* = −0.234, *p* = 0.000). There was also a significant positive correlation between improvements in biopsychosocial beliefs and pain neurophysiology knowledge post-intervention (*r* = 0.152, *p* = 0.031).

**Conclusion:**

Online educational interventions may enhance physiotherapists’ knowledge and shift attitudes and beliefs towards a biopsychosocial treatment orientation for LBP.

**Clinical implications:**

Incorporating concise, theory-informed online educational modules into continuing professional development may bridge knowledge gaps and facilitate the translation of biopsychosocial pain management strategies into routine physiotherapy practice.

## Introduction

The prevalence of chronic low back pain (LBP) worldwide is still on the rise, possibly because of mismanagement and inadequate treatment approaches that fail to recognise and manage the cognitive, psychological and social factors associated with non-specific chronic LBP (NSCLBP) (Bourne, Machado & Nagel 2014; Darlow et al. 2012).

Contemporary clinical practice guidelines for managing NSCLBP recommend a shift from the traditional biomedical model – focused on identifying and treating tissue pathology – towards a biopsychosocial orientation framework. This transition reflects growing recognition that biological factors alone cannot fully explain the persistence of pain and disability (Cowell et al. 2019; Meroni et al. 2021). Patients’ knowledge, attitudes and beliefs about pain, including misconceptions about injury, fear-avoidance behaviours and catastrophising, have been shown to influence symptom chronicity and treatment outcomes (Cowell et al. 2019; Meroni et al. 2021). Evidence indicates that limited pain knowledge and negative beliefs are associated with greater disability and poorer recovery (Tarimo & Diener 2017), while clinicians’ own biomedical-oriented attitudes can affect advice, activity recommendations and sick-leave practices (Gardner et al. 2017; Mukoka, Olivier & Ravat 2019; Lotia & Sheth 2021). Accordingly, a biopsychosocial approach is essential to address maladaptive cognitions and behaviours, enhance patient understanding and improve functional outcomes.

Recent studies have increasingly recommended the use of a systematic method of stratifying patients into clinically relevant sub-groups at low, medium and high-risk for transitioning to chronicity based on psychosocial factors and the tailoring of treatment according to their risk (Al Zoubi et al. 2019; Beneciuk et al. 2019). Reduced pain intensity, more targeted use of healthcare resources and a reduction in absenteeism were among the benefits found in a trial comparing stratified care with current best practice guidelines (Foster et al. 2014).

In African populations, contextual factors are widely recognised as key determinants of clinicians’ adherence to clinical practice guidelines, particularly those promoting biopsychosocial approaches to NSCLBP management (Ahenkorah et al. 2019). Among these contextual influences, the knowledge, attitudes and beliefs of healthcare providers themselves constitute a critical factor, as they directly shape clinicians’ readiness and ability to implement guideline-recommended psychosocial strategies in routine practice (Fitzgerald et al. 2018; Synnott et al. 2016). Limited training in recognising and addressing psychosocial contributors to NSCLBP continues to hinder effective implementation, highlighting the need for improved clinician education in pain science and biopsychosocial care (Fitzgerald et al. 2018; Synnott et al. 2016).

Strong evidence demonstrates that healthcare professionals’ knowledge, attitudes and beliefs directly influence clinical decision-making and treatment selection for LBP, with biomedical-oriented beliefs consistently associated with poorer adherence to evidence-based guidelines (Alshehri et al. 2020; Cowell et al. 2019; Gardner et al. 2017). Studies conducted in Europe, Asia, the Middle East and Australasia report persistent biomedical treatment orientations among physiotherapists and other healthcare professionals, despite growing endorsement of the biopsychosocial model (Alhowimel et al. 2021, 2022; Mikamo & Takasaki 2021; Rufa et al. 2022). For example, Alhowimel et al. (2021, 2022) identified moderate pain neurophysiology knowledge and a tendency towards biomedical beliefs among physiotherapists, which were associated with higher fear-avoidance messaging and less guideline-concordant care. Similarly, Mikamo and Takasaki (2021) reported that clinicians with stronger biomedical orientations were more likely to recommend rest, imaging and activity restriction, while Cowell et al. (2019) and Gardner et al. (2017) demonstrated that clinicians trained in a biopsychosocial framework achieved superior long-term reductions in pain and disability, even among patients who had previously failed conservative or surgical interventions. Collectively, these findings highlight substantial variability in clinician knowledge and treatment orientation across healthcare systems and cultural contexts, highlighting the importance of region-specific investigation. To date, however, no published studies have examined the knowledge, attitudes, beliefs or treatment orientations of clinicians managing LBP within the South African context. Given South Africa’s distinct healthcare structure, resource constraints and high musculoskeletal disease burden, locally derived evidence is necessary to support educational strategies and promote consistent, high-quality biopsychosocial care.

Effective management of LBP within a biopsychosocial framework requires that physiotherapists possess both a robust understanding of pain neurophysiology and the ability to apply risk stratification and biopsychosocial approaches in clinical practice. Translating this knowledge into routine care remains challenging because of barriers such as limited access to structured training, time constraints and the resource-intensive nature of traditional postgraduate education (Beneciuk & George 2015; Beneciuk et al. 2019). Online education offers a promising solution, providing scalable, flexible and cost-effective access to standardised, evidence-based content tailored to individual learning needs (Cook, Levinson & Garside 2010). Asynchronous formats guided by Cognitive Load Theory (CLT) enable complex material to be delivered in segmented, self-paced modules, reducing cognitive overload and enhancing retention (Ahmed 2024). Despite advances in contemporary pain science, a persistent knowledge-practice gap remains within physiotherapy, with evidence indicating that many clinicians continue to endorse inaccurate pain concepts and encounter significant barriers to implementing biopsychosocial care despite formal training (Smart 2023; Van Dijk et al. 2023; Wilson et al. 2025). Accordingly, our study investigated the effectiveness of a brief webinar series in enhancing physiotherapists’ pain-related knowledge and promoting a shift from predominantly biomedical orientations towards a patient-centred biopsychosocial model of care.

## Research methods and design

### Study design and setting

This is a quantitative, pre-post online survey-based, quasi-experimental study. The Template for Intervention Description and Replication (TIDieR) framework was followed in the reporting of the educational intervention.

### Participants and sampling

Qualified physiotherapists in South Africa who completed the ‘Implementing a Risk Stratification Model for Low Back Pain’ three-part webinar series and were members of the South African Society of Physiotherapy (SASP) at the time of data collection were included in our study. A convenience sampling method was employed, enrolling consenting participants who voluntarily registered for and completed the webinar series. Convenience sampling is a non-random method of selecting participants who are easiest to access, in this case, attending the webinars, and is used when quick, cost-effective data collection is needed.

Our study sample consisted of participants who completed both the pre- and post-webinar questionnaires. Data were available from 536 participants, all of whom attended the webinars. Of these, the first 320 participants (from 2016 to 2017) completed only the demographic questionnaire and the pre-intervention PABS-PT, as the pre-webinar NPQ was not introduced until 2018. All data collected prior to the intervention, including 536 completed demographic questionnaires, 536 completed PABS-PT questionnaires and 216 completed NPQs, were used to describe the demographic characteristics and baseline knowledge, attitudes and beliefs of the physiotherapists. For the analysis of intervention effects, only complete data sets, including 521 paired pre- and post-intervention questionnaires for the PABS-PT and 201 paired pre- and post-intervention NPQs, were included to compare participants’ scores before and after the webinar.

### Educational protocol

The educational intervention was a three-part webinar series designed to improve clinicians’ understanding and application of a risk stratification model for LBP management, using the STarT Back Screening Tool (SBST). The programme emphasised the biopsychosocial (BPS) model and evidence-based management approaches matched to low-, medium- and high-risk patient categories (Table 1).

**TABLE 1:** Overview of the educational intervention: ‘Implementing a risk stratification model for low back pain: A three-part webinar series’.

Session 1 (1-h duration)	Session 2 (2-h duration)	Session 3 (3-h duration)
Biopsychosocial approach to pain management Risk stratification – prognosis based Matched treatment pathways Use of STarT back screening tool Use of outcomes measures for pain Patient specific functional scale to measure activity and participation changes	Therapeutic alliance Communication skills Education based interventions including pain neuroscience Activity based interventions including graded exercises and activity Graded exposure Goal setting Mechanisms underpinning manual therapy interventions	
-	Clinical interventions appropriate for the management of low- and medium-risk low back pain patients	Clinical interventions appropriate for the management of high-risk low back pain patients

The educational programme was underpinned by principles of pain neuroscience education (PNE) (Louw et al. 2011; Moseley & Butler 2017), grounded in the biopsychosocial model of pain (Engel 1977). The webinars aimed to help participants reconceptualise pain as a protective output of the nervous system rather than a direct indicator of tissue damage, with the intention of fostering more adaptive beliefs and reducing fear-avoidance behaviours. The learning design drew on adult learning theory (Knowles, Holton III & Swanson 2014) and experiential learning principles (Kolb 1984), incorporating interactive discussions, clinical case examples and opportunities for reflection to facilitate the integration of new knowledge into participants’ clinical reasoning and practice.

#### Format and delivery

The webinar series was delivered online using PowerPoint presentations. Participants were able to engage with the presenters, ask questions and raise discussion points. The webinar was structured into three parts to facilitate participants’ cognitive processing and to present the content in a manageable format. The design applied principles of cognitive load theory, presenting content in manageable, structured segments to optimise learning. The total duration was 5 h, divided into three sessions over 3 weeks. Following the intervention, participants were presented with a detailed written case study and accompanying follow-up questions designed to challenge their clinical decision-making, including their treatment recommendations and selection of educational strategies. Session recordings were provided to participants to support self-directed, asynchronous review and facilitate the consolidation of knowledge.

#### Instructors and materials

Two physiotherapists with postgraduate qualifications (master’s and doctoral degrees) in neuromusculoskeletal physiotherapy developed and presented the webinars. Both had extensive experience in postgraduate education and clinical practice and were recognised as subject-matter experts. Supplementary resources included reading lists and validated clinical tools (SBST, Patient-Specific Functional Scale).

#### Fidelity and quality assurance

Content development was collaborative and guided by current clinical guidelines for LBP management. Both presenters reviewed each other’s materials for accuracy and consistency.

### Instrumentation and outcome measures

A demographic questionnaire, the Neurophysiology of Pain Questionnaire (NPQ) and the Pain Attitudes and Beliefs Scale for Physiotherapists (PABS-PT) and were used for the data collection.

The NPQ was formulated to evaluate how an individual conceptualises the biological processes underpinning pain (Catley, O’Connell & Moseley 2013) and has been used previously to determine the impact of brief educational interventions in clinical practice (Fitzgerald et al. 2020). Catley et al. made use of the revised NPQ consisting of 13 true or false questions. Correct responses were allocated one point, whereas incorrect or undecided responses scored no points. A higher score reflects a greater understanding of the neurophysiology of pain.

The PABS-PT was constructed to distinguish between the biomedical and biopsychosocial treatment orientations of clinicians (Bishop 2010). The version of the PABS-PT questionnaire used for our study consisted of 31 questions and took approximately 10 min to complete (Ostelo et al. 2003). Respondents indicated the level to which they agree or disagree with each statement on a Likert scale. Summation of the responses corresponding to the items within each subscale provides the total scores for either the biomedical subscale or biopsychosocial subscale. Higher scores on each subscale will indicate a stronger or dominant treatment orientation, that is a higher score on the biomedical subscale indicates a prevailing preference towards a biomedical treatment approach or orientation (Bishop 2010). During the development of the 31-item PABS-PT by Ostelo et al. (2003), items 19 and 29 reflected on both the biomedical and biopsychosocial subscales and were therefore excluded from the data analysis of our study (Ostelo et al. 2003).

### Data extraction

Data for our study were collected using structured questionnaires and managed through the Research Electronic Data Capture (REDCap) platform hosted by the University of the Witwatersrand, Johannesburg.

The dataset was exported from REDCap into Microsoft Excel and securely stored in a password-protected folder. Records from participants who did not consent to the use of their data were excluded. Data were de-identified by assigning unique study identification numbers, and access to identifiable data was restricted to authorised personnel.

Participants received an electronic demographic survey and links to pre-webinar questionnaires via email 48 h before the webinar series. Links to post-intervention PABS-PT and NPQ questionnaires were distributed immediately after the intervention. Between 2016 and 2018, participants completed only the demographic and pre-intervention PABS-PT questionnaires; from 2018 onward, the pre-intervention NPQ was also included. Data were stored in Research and Electronic Data Capture (REDCap) software program, where unique identification numbers automatically linked pre- and post-intervention responses anonymously.

### Data analyses

All collected data were used to analyse the demographic characteristics and baseline knowledge, attitudes and beliefs of the physiotherapists who participated in our study. Complete sets of data included those participants who completed all the pre- and post-intervention questionnaires. Only the complete sets of questionnaires were used to compare pre- and post-intervention scores of the participants.

The quantitative data obtained from the questionnaires were exported from the REDCap tools hosted at the University of the Witwatersrand (Harris et al. 2009) and cleaned, organised and captured in Microsoft Excel spreadsheets. The STATA statistical analysis package version 17.0 standard edition was used for analysis.

The biomedical attitudes, beliefs and treatment orientations were observed from the scores obtained from the 20-item PABS-PT biomedical subscale. Attitudes, beliefs and treatment orientation with more biopsychosocial or behavioural qualities were assessed using the 9-item PABS-PT biopsychosocial subscale. The NPQ was scored out of 13, with one point awarded for each accurate response.

Descriptive statistics were generated to determine the demographic profile, baseline attitudes and beliefs, and knowledge regarding LBP of the cohort of physiotherapists. Means and standard deviation were used for continuous data, and frequencies and percentages for categorical data. The data from the PABS-PT and NPQ questionnaires were compared before and after the online education programme using dependent *t*-tests. The association between the knowledge of participants (as per the NPQ) and their attitudes and beliefs (as per the PABS-PT), as well as the association between the knowledge, attitudes and beliefs were compared using Pearson correlation analyses.

### Ethical considerations

Ethical clearance was granted by the University of the Witwatersrand Human Research and Ethics Committee. The ethical clearance number is M210299. Each participant provided written informed consent for their information to be used for the purposes of our study. Anonymity was ensured and maintained by automatically allocating de-identified study identity numbers to each participant to link the pre-webinar data to the post-webinar data. No captured information or data were shared with the participants. All data has been used solely for the purpose intended for in our study.

## Results

**FIGURE 1:**
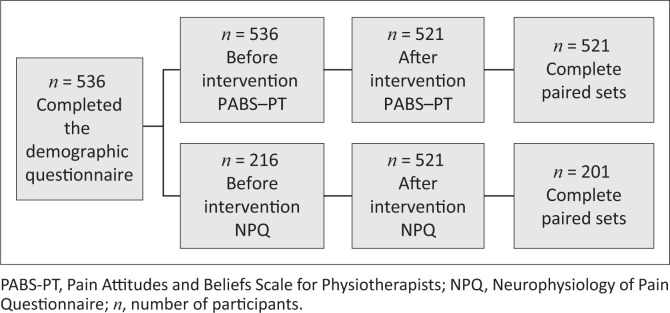
Summary of study participants.

A total of 536 physiotherapists completed both the demographic questionnaire and the PABS-PT questionnaire prior to the educational intervention (Figure 1). Only 216 participants (40.3%) returned the completed pre-intervention NPQ, as this requirement was introduced midway through data collection. In contrast, participants were asked to complete the post-webinar NPQ from the start of data collection, resulting in a larger number of post-intervention NPQ responses. Following completion of the webinar series, 521 participants (97.2%) submitted both a completed PABS-PT and NPQ. Consequently, pre- and post-intervention comparisons were conducted using 521 paired PABS-PT datasets and 201 paired NPQ datasets.

Of the 536 participants, most were aged 31–40 years (36.8%), followed by < 30 years (27.1%) (Table 2). The majority were female (86.8%). Most participants obtained their undergraduate qualification between 2000 and 2009 (35.0%). The highest qualification held by most respondents was a bachelor’s degree (84.5%), with smaller proportions holding master’s (11.8%) or doctoral degrees (1.1%).

**TABLE 2:** Participant demographical characteristics.

Demographical characteristic	*n*	%
**Age (years) (*n* = 536)**		
≤ 30	145	27.05
31–40	197	36.75
41–50	106	19.78
≥ 50	88	16.42
**Gender (*n* = 536)**		
Female	465	86.75
Male	71	13.25
**Undergraduate qualification (*n* = 535)**		
Obtained before 1979	27	5.05
1980–1989	66	12.34
1990–1999	108	20.19
2000–2009	187	34.95
2010–2018	147	27.48
**Highest qualification (*n* = 536)**		
Bachelor’s degree	453	84.51
Master’s degree	63	11.75
Doctoral degree	6	1.12
Other: Diploma	14	2.61
**Field of clinical practice (*n* = 536)**		
In-hospital	213	39.74
Musculoskeletal outpatient	491	91.60
Musculoskeletal sports	249	46.46
Paediatrics	86	16.04
Neurology	107	19.96
Public health	11	2.05
Other	76	14.18
**Post-graduate training (*n* = 536)**		
OMT	229	42.72
SPT	33	6.16
Pain: Whole course	18	3.36
Pain: Principles of pain	87	16.23
Pain: Communication skills	50	9.33
Pain: Interventions	38	7.09
Pain: Electives	36	6.72
Other	89	16.60
None	178	33.21

OMT, orthopaedic manipulative therapy; SPT, sports physiotherapy; *n*, number of participants; %, percentage.

A high number of participants (66.8%) had undergone some form of post-graduate training where pain neuroscience was a component of the material taught. Common fields of clinical practice included musculoskeletal outpatient care (91.6%), sport (46.5%) and in-hospital settings (39.7%), with fewer working in neurology (20.0%), paediatrics (16.0%), public health (2.1%) or other areas (14.2%).

**FIGURE 2:**
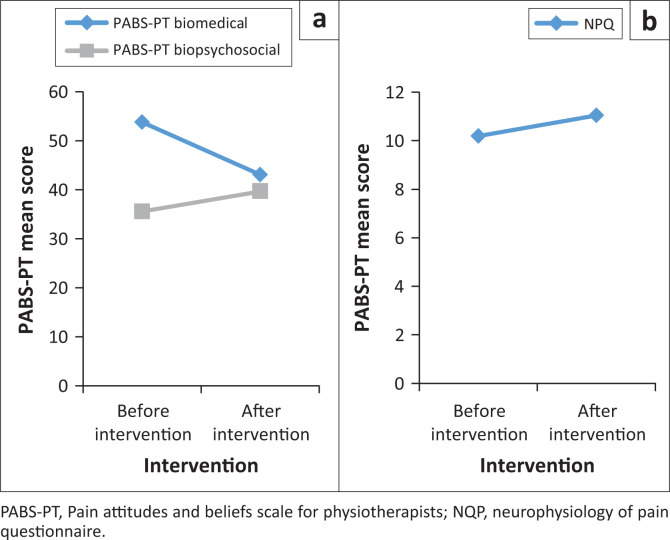
Pain attitudes and beliefs scale for physiotherapists and neurophysiology of pain questionnaire mean scores before and after intervention.

The results from the PABS-PT and NPQ revealed significant mean score changes in the PABS-PT and NPQ outcome measures (Figure 2 and Table 3).

**TABLE 3:** Mean difference values and analysis of variance of Pain Attitudes and Beliefs Scale for Physiotherapists and Neurophysiology of Pain Questionnaire.

Outcome measure	Before intervention			After intervention		Mean difference		*t*	*p*-value
	*n*	Mean ± s.d.	Confidence interval (95%)	Mean ± s.d.	Confidence interval (95%)	Mean ± s.d.	Confidence interval (95%)		
PABS-PT biomedical subscale (20 items)	521	53.74 ± 12.80	52.53, 54.74	43.04 ± 11.63	42.04, 44.04	10.60 ± 10.44	9.70, 11.50	23.16	0.00
PABS-PT biopsychosocial subscale (9 items)	521	35.52 ± 4.65	35.14, 35.94	39.62 ± 4.70	39.22, 40.03	−4.09 ± 5.09	−4.53, -3.65	−18.32	0.00
NPQ (13 items)	201	10.19 ± 1.78	9.92, 10.41	11.04 ± 1.64	10.79, 11.25	−0.86 ± 1.65	−1.09, -0.63	−7.35	0.00

PABS-PT, Pain Attitudes and Beliefs Scale for Physiotherapists; NPQ, Neurophysiology of Pain Questionnaire; *n*, number of participants; s.d., standard deviation.

Analysis of pre- and post-intervention scores revealed significant changes in physiotherapists’ attitudes, beliefs and pain knowledge. On the PABS-PT biomedical subscale, participants’ scores decreased by 8.8% from a mean of 53.74 ± 12.80 (95% CI: 52.53–54.74) before the intervention to 43.04 ± 11.63 (95% CI: 42.04–44.04) after the intervention, representing a mean difference of 10.60 ± 10.44 (95% CI: 9.70–11.50, *t* = 23.16, *p* < 0.001). A reduction in the biomedical subscale score reflects an improvement, indicating a shift away from a purely biomedical orientation towards a more contemporary, biopsychosocial approach. Conversely, the PABS-PT biopsychosocial subscale scores demonstrated a 7.6% increase from 35.52 ± 4.65 (95% CI: 35.14–35.94) to 39.62 ± 4.70 (95% CI: 39.22–40.03), with a mean difference of −4.09 ± 5.09 (95% CI: −4.53 to −3.65, *t* = −18.32, *p* < 0.001). An increase on this subscale reflects improvement, indicating stronger endorsement of biopsychosocial principles. Similarly, NPQ scores improved by 6.6% from 10.19 ± 1.78 (95% CI: 9.92–10.41) to 11.04 ± 1.64 (95% CI: 10.79–11.25), with a mean difference of −0.86 ± 1.65 (95% CI: −1.09 to −0.63, *t* = −7.35, *p* < 0.001), demonstrating a significant increase in pain neurophysiology knowledge.

Our study found a weak but significant negative relationship between changes in PABS-PT biomedical and biopsychosocial subscales scores (*r* = −0.2326, *p* = 0.0000). Significant improvements in biopsychosocial attitudes were positively associated with increases in NPQ scores (*r* = 0.1519, *p* = 0.0314), while reductions in biomedical attitudes were inversely correlated with NPQ improvements (*r* = −0.1215, *p* = 0.0766), suggesting that shifts in physiotherapists’ beliefs are closely linked to their understanding of pain neurophysiology.

**FIGURE 3:**
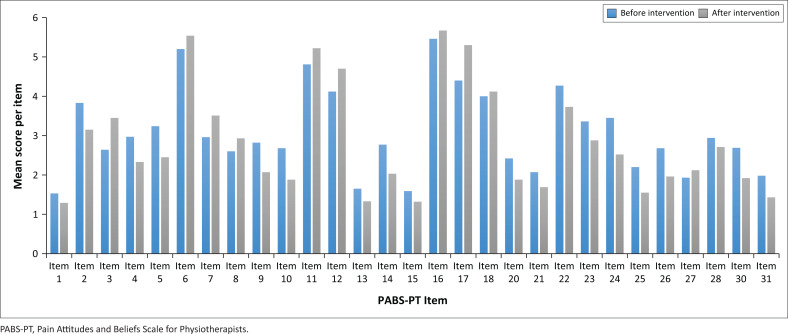
Mean score per item on Pain Attitudes and Beliefs Scale for Physiotherapists. Full description of each item available in Online Appendix 1 Table 1-A1. Items 19 and 29 reflect on both Biomedical and Biopsychosocial subscales and were therefore excluded from our study data analysis.

The mean scores per item on the PABS-PT before and after the webinar are reflected in Figure 3. Before the intervention, physiotherapists’ responses on the PABS-PT reflected an apparent biomedical orientation towards LBP. Several biomedical subscale items demonstrated relatively high scores, indicating moderate to strong agreement with statements emphasising caution, activity limitation and the presence of tissue damage (e.g. ‘Reduction of daily physical exertion is a significant factor in treating back pain’; ‘Not enough effort is made to find the underlying organic causes of back pain’). Conversely, biopsychosocial subscale items generally yielded lower scores, reflecting limited endorsement of biopsychosocial perspectives, particularly regarding the influence of psychosocial factors and patient perceptions on pain and functional outcomes (e.g. ‘A patient suffering from severe back pain will benefit from physical exercise’; ‘Therapy may have been successful even if pain remains’).

Conversely, biopsychosocial subscale scores increased, indicating enhanced recognition of psychosocial, cognitive and behavioural contributions to pain. Notably, agreement with ‘Mental stress can cause back pain even in the absence of tissue damage’ increased from 5.20 ± 1.04 to 5.54 ± 0.96, and ‘The way patients view their pain influences the progress of the symptoms’ increased from 5.46 ± 0.85 to 5.67 ± 0.73. Internal consistency was high for the biomedical subscale, both pre- and post-intervention (Cronbach’s α = 0.865–0.879), whereas the biopsychosocial subscale demonstrated moderate reliability (Cronbach’s α = 0.524 pre-intervention; 0.540 post-intervention), consistent with prior validations of the PABS-PT.

Following the intervention, physiotherapists’ beliefs regarding back pain exhibited a discernible shift from predominantly biomedical orientations towards greater acknowledgement of biopsychosocial factors. On the biomedical subscale, mean scores decreased across many items, indicating a reduction in distinct biomedical perspectives. For instance, agreement with statements such as ‘Back pain sufferers should refrain from all physical activity to avoid injury’ declined from 1.53 ± 0.88 pre-intervention to 1.29 ± 0.78 post-intervention, while endorsement of ‘Pain is a nociceptive stimulus, indicating tissue damage’ decreased from 2.68 ± 1.50 to 1.88 ± 1.30.

**FIGURE 4:**
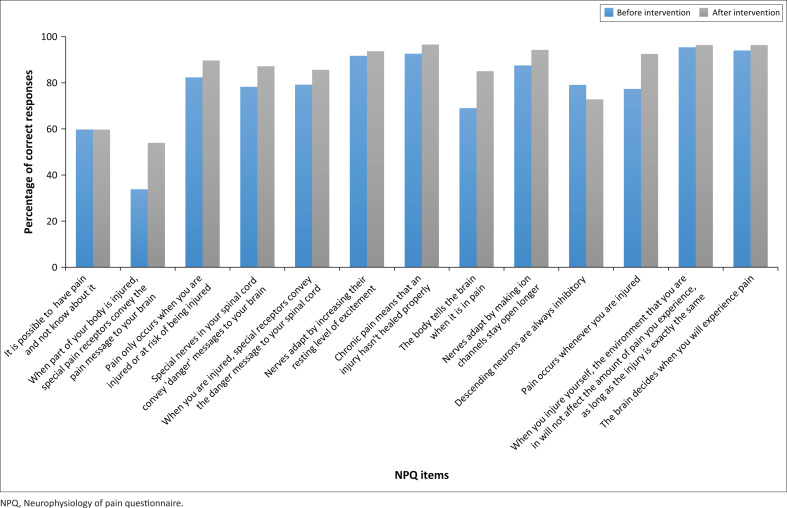
Percentage of correct responses per item on neurophysiology of pain questionnaire.

The percentage of correct answers per item on the NPQ before and after the webinar are reflected in Figure 4.

At baseline, participants’ knowledge was limited for several items, notably Item 2 (‘When part of your body is injured, special pain receptors convey the pain message to your brain’), which was correctly answered by only 33.8% of participants. Moderate performance was observed for Items 1 (59.7%) and 8 (69.0%), indicating gaps in understanding of pain perception and neural mechanisms.

Following the intervention, correct responses on the NPQ increased across most items, with the largest improvement observed for those with low baseline scores. Item 2 improved from 33.8% to 53.9%, Item 8 from 69.0% to 85.0% and Item 11 from 77.3% to 92.5%. Items with high baseline scores (Items 12 and 13) exhibited minimal change, consistent with ceiling effects. These findings indicate that the intervention substantially enhanced participants’ understanding of neurophysiological pain mechanisms, particularly in areas of prior low knowledge. Item-level internal consistency, as measured by Cronbach’s alpha, ranged from 0.50 to 0.57 before the intervention and 0.51 to 0.58 after, with overall NPQ reliability increasing slightly from 0.559 to 0.567.

## Discussion

The findings of our study indicate that participation in a 5-h pain neuroscience webinar significantly enhanced participants’ understanding of pain mechanisms. Moreover, the intervention promoted a discernible shift in treatment orientation, moving from a predominantly biomedical approach towards a more comprehensive biopsychosocial model of care. These outcomes align with international evidence indicating that targeted educational interventions can effectively modify clinicians’ conceptualisation of pain and promote alignment with contemporary, evidence-based guidelines (Bareiss, Nare & McBee 2019; Leysen et al. 2021; Springer, Gleicher & Hababou 2018).

The integration of psychological and social elements within the assessment and management of LBP is fundamental to the biopsychosocial model of care, which has been associated with enhanced patient outcomes and reduced healthcare utilisation (Childs et al. 2015; Foster et al. 2018). Clinicians’ attitudes and beliefs impose a substantial influence on patients’ perceptions, recovery expectations, and functional outcomes (Darlow et al. 2012; Gardner et al. 2017). Practitioners exhibiting stronger biomedical orientations are more likely to advise activity restriction and utilise passive modalities that are incongruent with current guideline recommendations (Alshehri et al. 2020; Meroni et al. 2021). Conversely, those demonstrating biopsychosocial orientations are more inclined to employ active strategies such as graded exercise, patient education and cognitive-behavioural principles (Oliveira et al. 2018).

Despite substantive advances in conceptualising pain as a complex experience influenced by nervous system plasticity and biopsychosocial factors, a persistent knowledge–practice gap exists in physiotherapy.

Cross–sectional evidence indicates that while physiotherapists report high perceived competence in pain science education (PSE), a significant proportion endorse inaccurate pain science concepts, despite formal training, and report multiple implementation barriers, including patient expectations, time constraints and clinician–specific challenges in addressing maladaptive beliefs and behaviours (Wilson et al. 2025). Systematic reviews further demonstrate that although physiotherapists may be generally aware of biopsychosocial principles, knowledge, skills, attitudes, environmental context, confidence and patient expectations continue to limit effective integration of these approaches in chronic pain care (Van Dijk et al. 2023).

These challenges are exacerbated by systemic educational gaps that leave many graduates underprepared for complex pain management, as pain neuroscience and biopsychosocial frameworks are inconsistently integrated into curricula (Smart 2023). The evidence emphasises that improving pain management in physiotherapy requires the dissemination of contemporary pain science that aligns clinician beliefs and behaviours with best–practice biopsychosocial care.

Our study sample comprised 536 physiotherapists, representing a robust cohort relative to similar international investigations (Alhowimel et al. 2021; Mikamo & Takasaki 2021; Petit et al. 2019). While the sample size is a strength, the use of a convenience sampling strategy, a non-probability method, introduces inherent limitations. Participants were recruited based on their accessibility and voluntary election to attend the webinars (Andrade 2021). Although this approach is a pragmatic necessity in educational research, it carries an increased risk of selection bias. Clinicians who self-select into PNE may differ systematically from the broader professional population regarding baseline knowledge, motivation or existing professional interests (Andrade 2021). Consequently, the representativeness of this sample may be compromised, potentially limiting the external validity and generalisability of the findings to the wider physiotherapy community.

Baseline PABS-PT and NPQ scores indicated a relatively favourable starting point for this cohort, likely reflecting prior postgraduate exposure or musculoskeletal specialisation. However, the statistically significant post-intervention shifts suggest that structured online education can further enhance alignment with biopsychosocial management strategies, even among clinicians with moderate-to-high baseline knowledge.

This observation is consistent with literature demonstrating that focused interventions can influence practice orientation regardless of prior experience levels (Domenech et al. 2011; Overmeer et al. 2011; Rufa et al. 2022).

The magnitude of change observed in our study is consistent with outcomes reported in comparable international interventions. For instance, Saracoglu et al. (2021) reported a 6% – 7% increase in biopsychosocial orientation and concomitant reductions in biomedical beliefs among physiotherapists following a structured online module, despite relatively high baseline scores. Similarly, Bareiss et al. (2019) demonstrated significant improvements in PABS-PT and NPQ scores among physiotherapy students after short-format, interactive pain science workshops. Zimney et al. (2018) reported that brief online PNE interventions could yield significant improvements in clinician knowledge, attitudes and confidence in managing NSLBP. The knowledge gains observed in this study (6.6%) further support the efficacy of short-format interventions for practising clinicians (Cox, Louw & Puentedura 2017; Wassinger 2021). These results suggest that online education serves to reinforce and consolidate pre-existing knowledge, bridging the gap between undergraduate training and advanced clinical reasoning (Mikamo & Takasaki 2021; Fourré et al. 2022).

These convergent findings suggest that even short, well-structured educational interventions can produce meaningful cognitive and attitudinal change among practising clinicians, particularly when designed to engage learners actively and leverage principles such as Cognitive Load Theory (Ahmed 2024). A critical advantage of online education is its potential to overcome traditional barriers to widespread pain neuroscience training.

Conventional postgraduate courses are often resource-intensive, requiring travel, extended time commitments and high financial costs, which restricts participation and limits equitable access (Beneciuk & George 2015; Beneciuk et al. 2019). In contrast, online modules can be delivered flexibly, allowing self-paced learning without substantial incremental cost. This is particularly relevant in low- and middle-income settings, where access to formal postgraduate education may be limited, and for clinicians balancing clinical responsibilities with professional development. Additional advantages include accessibility for geographically dispersed clinicians, opportunities for repeated review and reinforcement, and the potential to integrate interactive elements, such as quizzes and case-based scenarios to strengthen application in clinical reasoning (Hung et al. 2024). Compared with synchronous approaches, asynchronous learning maintains comparable outcomes while increasing learner autonomy and engagement (Hung et al. 2024). By leveraging these advantages, online postgraduate pain education represents a feasible and effective strategy to strengthen physiotherapists’ biopsychosocial competencies, directly addressing the objectives of our study to evaluate the impact of an online, modular PNE intervention on physiotherapists’ knowledge, attitudes and beliefs in LBP management.

Nevertheless, challenges remain for large-scale implementation. Engagement and completion rates can vary, and ensuring interactivity and application to clinical reasoning is critical for translating knowledge gains into practice (Cook et al. 2010; Hung et al. 2024). Furthermore, sustained reinforcement, through repeated modules, mentorship or integrated clinical practice exercises, may be necessary to consolidate learning and support behavioural change. Cost-effectiveness analyses of online interventions remain sparse, but preliminary evidence suggests that the reduced logistical and personnel costs associated with asynchronous delivery could make online education a viable strategy for broad-scale dissemination of contemporary pain science (Ahmed 2024; Hung et al. 2024).

Correlation analyses revealed a weak negative association between biomedical and biopsychosocial subscales, suggesting that a reduction in biomedical beliefs was accompanied by a corresponding increase in biopsychosocial orientation. Moreover, a measurable positive correlation emerged between enhanced pain knowledge and biopsychosocial beliefs, aligning with previous findings indicating that improved pain knowledge and comprehension are linked to greater adherence to evidence-based, patient-centred care (Christe, Darlow & Pichonnaz 2021; Fitzgerald et al. 2018; Mikamo & Takasaki 2021). Collectively, these findings reinforce the premise that conceptual understanding of pain mechanisms is integral to the adoption of contemporary management paradigms for CLBP. Whether this conceptual shift leads to meaningful behavioural changes remains uncertain.

The positive effects observed in our study likely derive from pedagogical design, which engaged clinicians in the conceptual foundations of contemporary pain science. The inclusion of content addressing nervous system plasticity, cognitive and psychosocial contributors to pain, and the limitations of strictly biomedical approaches appears to have facilitated meaningful conceptual shifts. These mechanisms are in line with prior research indicating that comprehension of pain mechanisms is linked to stronger endorsement of biopsychosocial principles and evidence-based treatment behaviours (Christe et al. 2021; Fitzgerald et al. 2018).

### Clinical implications

Pain is increasingly recognised as a multifactorial construct influenced by an interplay of biological, psychological and social factors. Enhancing clinicians’ understanding of these dimensions promotes a more holistic, empathetic and effective approach to patient care. The present findings contribute to the growing body of evidence demonstrating that concise, cost-effective and attainable educational interventions can produce measurable improvements in physiotherapists’ knowledge and belief systems. Such initiatives have the potential to advance clinical reasoning, foster adherence to international practice guidelines, and ultimately improve patient outcomes while mitigating the societal burden associated with chronic pain (Foster et al. 2018; Lemmers et al. 2022). Future research should examine whether these positive cognitive and attitudinal changes translate into sustainable behavioural adaptations and improved functional outcomes for patients with CLBP.

### Limitations and future research considerations

Our study is subject to several methodological limitations. First of all, reliance on self-reported measures may introduce response and social desirability biases. The absence of a control group constrains causal inference regarding the observed effects of the intervention. Although post-intervention improvements were evident, the durability of these changes over time and their translation into clinical behaviour were not assessed.

Although the sample size is a notable strength, the employment of a convenience sampling strategy, predicated on participants’ voluntary attendance, introduces potential selection bias and may compromise the representativeness of the cohort, thereby limiting the external validity and generalisability of the findings.

Furthermore, recruitment through professional networks may have yielded a sample predisposed towards evidence-based and musculoskeletal practice, thereby limiting generalisability. Future investigations should adopt controlled, longitudinal designs incorporating objective behavioural and patient-targeted outcomes.

Comparative analyses of varying educational formats, durations and delivery modalities would further clarify optimal strategies for integrating PNE into continuing professional development frameworks. Establishing a direct link between enhanced clinician knowledge, treatment orientation and patient recovery trajectories remains a critical avenue for future research.

## Conclusion

Our study demonstrates that a brief online education programme can effectively enhance physiotherapists’ knowledge and foster a shift from biomedical to biopsychosocial treatment orientations for chronic LBP.

These findings highlight the modifiable nature of clinicians’ beliefs and underscore the potential of targeted, accessible education to promote evidence-based, person-centred physiotherapy practice. Broader implementation of such interventions may strengthen clinical reasoning, improve adherence to guidelines and ultimately optimise patient outcomes in chronic pain management.
